# Supplementary figures and images for: Linguistic Markers in Spontaneous Speech: Insights into Subjective Cognitive Decline (Review)
Source: Healthcare (Basel). 2025 Nov 13;13(22):2888. doi: 10.3390/healthcare13222888 (PMC12652931; doi:10.3390/healthcare13222888)

# PRISMA Flow Diagram

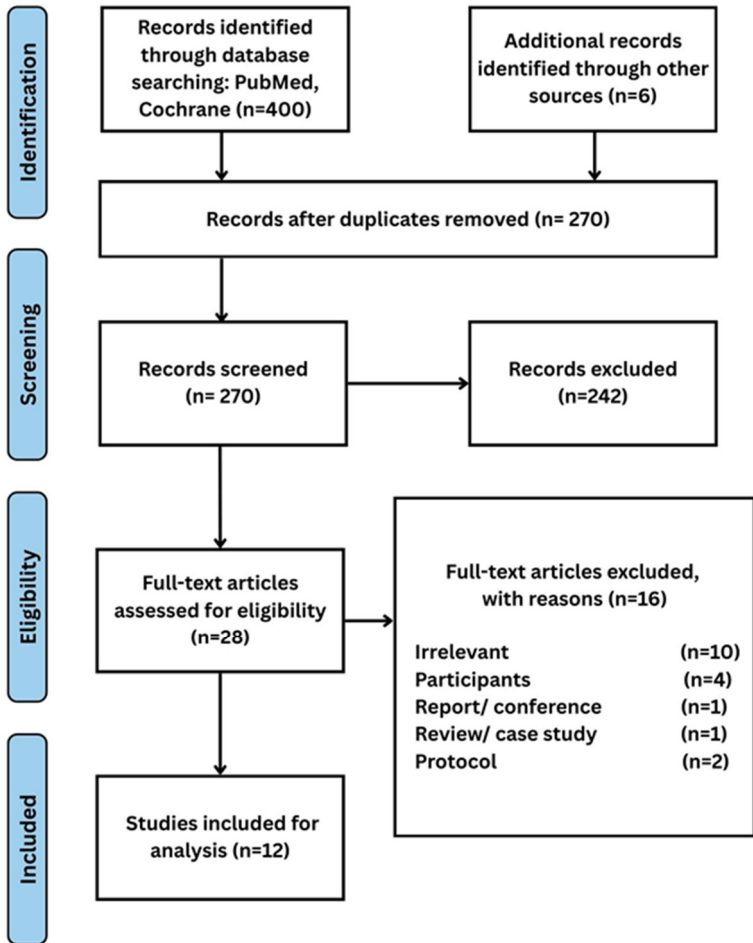

Supplement: Supplementary file 1 [file healthcare-13-02888-s001.zip › healthcare-3813960-supplementary.pdf]
